# Medical research production in native languages: A descriptive analysis of PubMed database

**DOI:** 10.5339/qmj.2024.21

**Published:** 2024-05-02

**Authors:** Abdullah Ashraf Hamad, Jaber H. Jaradat, Hamza K. Alsalhi, Ibraheem M Alkhawaldeh

**Affiliations:** 1 Faculty of Medicine, Menoufia University, Shibin El-Kom, Egypt Email: abdullah.hamad744@gmail.com; 2 Medical Research Group of Egypt, Negida Academy, Arlington, MA, USA; 3 Faculty of Medicine, Mutah University, Al-Karak, Jordan; 4 Faculty of Medicine, The Hashemite University, Zarqaa, Jordan

**Keywords:** Medicine, medical research, language, native language, mother tongue, disparities

## Abstract

Introduction: Language barriers in medicine can hinder effective communication, comprehension, and patient care. While English has emerged as the dominant language in global medicine, the importance of native languages should not be overlooked. This article aims to examine the extent of publishing in native languages by analyzing the PubMed database literature to gain further insights into the usage of native languages in medicine and medical research.

Methods: In December 2023, a comprehensive examination of the PubMed literature was conducted for each of the 55 registered languages. We searched for records published in each language (e.g., German[lang]) by applying language filters. Ethnologue provided data on the number of worldwide native speakers for each language, facilitating a comparative analysis.

Results: By December 2023, PubMed contained over 36 million publications, with 86.5% of them published in English. German, French, and Russian came after English, with over 700 thousand publications each. Among the languages analyzed, fourteen had fewer than 50 publications, nineteen had fewer than 100, twenty-two had fewer than 500, and twenty-five had fewer than one thousand publications. European languages were well-represented with thousands of publications each, while widely spoken languages such as Hindi and Arabic had limited representation.

Conclusion: The production of medical research in native languages reflects the attention given to native languages in medicine and medical education within each country. It is crucial to provide due attention to these language-related issues and explore strategies for including native languages in medicine to bridge the gaps in language and medicine.

## Introduction

Medicine is the milestone of maintaining and improving people’s health, and access to essential healthcare services is considered a part of human rights.^1^ However, language barriers can hinder effective communication, comprehension, and patient care.^2^ Choosing a foreign language as the medium of instruction in medical education significantly impacts students’ understanding, academic performance, and ability to communicate with patients and colleagues.^3^ Moreover, relying on a foreign language in medicine can significantly affect healthcare and public health.^4^

While English has emerged as the dominant language in global medicine and medical research, the importance of native languages in non-English-speaking countries should not be overlooked.^5,6^ Developed countries, often at the forefront of medicine and healthcare, primarily rely on their native languages. In contrast, developing countries tend to have a prevalence of foreign language-based medical practices, as highlighted in Hamad’s research.^6^ Furthermore, research contributions in native languages enrich the medical literature and enhance healthcare systems.^4,7,8^ This article aims to examine the extent of publishing in native languages to gain further insights into using native languages in medicine and medical research by analyzing the PubMed database literature.

## Methods

On December 5, 2023, we individually examined the PubMed literature for each of the 55 languages registered on PubMed. We applied the language filter to search for records published in each language (e.g., German[lang]). The languages were then sorted based on the publication count. Additionally, we illustrated the publication rates over time for the highly represented languages. To facilitate a comparison between languages, we included information on the number of native speakers worldwide for each language. We relied on Ethnologue, an evidence-based reference providing statistics and information on the living languages of the world, to determine the number of native speakers for each language.^9^ The number of native speakers for each language was defined as the number of users in all countries using L1 (first language), as reported on Ethnologue.

## Results

As of December 2023, over 36 million citations were published on PubMed. English accounted for 86.5% of all records published on PubMed, confirming its dominance in medical research. German and French followed at a distant second and third place, with 2.5% and 2.1% of publications, respectively. Our analysis encompassed a total of 55 individual languages, in addition to publications with undetermined languages and publications with multiple languages (Table 1).

Examining the publication counts by language, we found that English was the only language with over one million publications (32 million). German, French, and Russian came after English, with over 700 thousand publications each. On the other hand, languages such as Maori, Sanskrit, and Malay had the lowest publication counts, with only one record each. Furthermore, fourteen had fewer than 50 publications, nineteen had fewer than 100, twenty-two had fewer than 500, and twenty-five had fewer than one thousand publications. Less widely spoken European languages, like Czech, Danish, Swedish, and Norwegian, were well represented with thousands of publications each.

**Figure 1.**
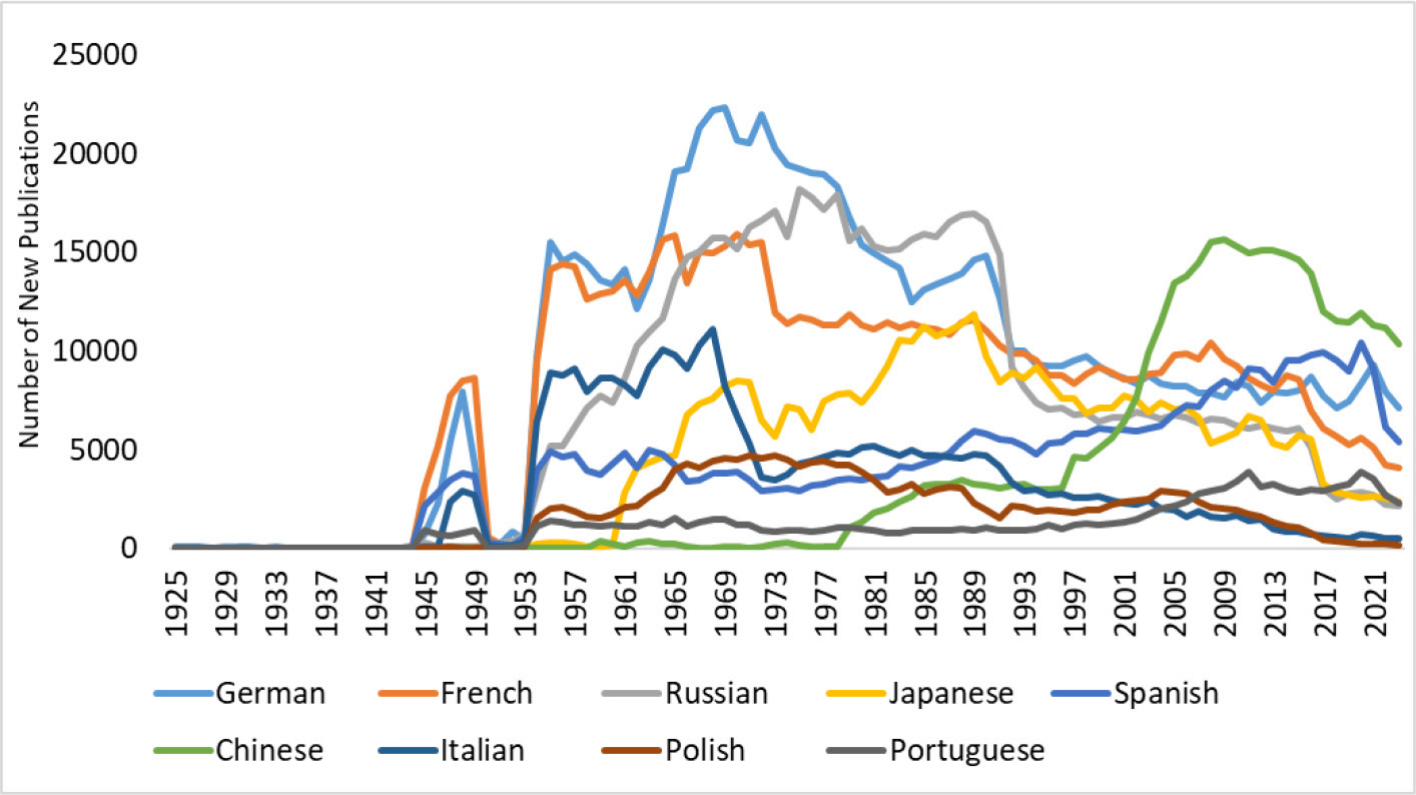
Number of new publications per year for highly represented languages in PubMed literature (from inception until 2023).

On the other hand, many other widely spoken languages, such as Hindi, Arabic, and Malay, had limited representation or were almost absent. The rate of publishing in English is increasing over time. For other highly represented languages, the rate of publications showed different patterns, with most of them exhibiting a relatively decreasing trend (Figure 1).

## Discussion

Over the years, research has played a crucial role in advancing medical sciences, providing the scientific community with the necessary tools to expand their discoveries. Researchers’ choice of publishing language is influenced by various factors, including their familiarity with a language, the extent of its usage in the field, and the desire to enrich the native language literature.^10^ Publishing in English, currently the most widely used language in medicine and research, offers advantages such as greater access to a broader audience and increased citation potential for authors seeking higher citation indexes.^11^ On the other hand, publishing in the native language of a country enriches its medical literature, enhances understanding of this literature, and improves public health education and promotion.^4,5^

To the best of our knowledge, this study represents the first analysis of the status of medical research production in native languages. We specifically chose to examine the PubMed database, as it is one of the leading and widely recognized medical databases, encompassing reputable journals with stringent indexing criteria.^12^ Additionally, PubMed accepts journals fulfilling the criteria, irrespective of the publication language.^12^

The language representation in our analysis reveals interesting patterns. European languages are well-represented, reflecting the attention given to native languages in these countries. However, there is a notable absence or limited representation of many widely spoken languages, such as Hindi and Arabic. In contrast, less commonly spoken languages like Czech, Swedish, and Finnish enjoy considerable representation in medical research. This observation aligns with Hamad’s study, which highlights the dependence on native languages in medical education in each country.^6^

One noteworthy finding is the growing presence of medical research in Chinese. This observation aligns with China’s experience and progress in modern medicine. Until the 1940s, China relied primarily on what is known as “Chinese traditional medicine”.^13^ However, internal and external conflicts had a detrimental impact on Chinese healthcare during that time. Following the establishment of the People’s Republic of China in 1949, the country took a different path in healthcare by paying attention to Western medicine.^14^ Despite slow economic growth, the Chinese healthcare system made significant improvements between 1950 and 1990, including doubling life expectancy and a substantial decline in infant mortality.^15^ China also transitioned from combating infectious diseases to addressing chronic non-communicable diseases, like other developed countries.^16^ Notably, this progress in medicine and medical education relied exclusively on the Chinese language, making it one of the most successful examples of mother-tongue-based education in the modern era. Starting from 1950, publications in the Chinese language exhibited a consistent upward trend, as observed in PubMed data. Also, China established several biomedical databases covering thousands of journals and millions of articles, significantly enriching the Chinese medical library.^17^ This emphasis on developing their own databases might explain the notable descending curve in the last decade, as researchers in China focus on utilizing and contributing to the Chinese databases rather than relying solely on global ones like PubMed.

The findings of this study shed light on the status of medical research production in native languages and the implications of language disparities in the field of medicine. The dominance of English in medical research publications is evident, with 86.5% of articles published on PubMed being in English. This reaffirms the global prominence of English as the lingua franca of scientific communication. However, it is crucial to recognize the significance of native languages in non-English-speaking countries and the potential benefits they bring to healthcare systems and medical literature. Further exploration is warranted to understand better the extent to which native languages are utilized in medical research and the potential impact this has on individuals’ health outcomes. For instance, incorporating native languages could make medical knowledge and literature more accessible and understandable to the general public.^5^ Future research efforts should aim to investigate the specific ways in which incorporating native languages in medical research can positively influence healthcare delivery, patient understanding, and overall health outcomes.

## Conclusion

The production of medical research in native languages reflects the attention given to native languages in medicine and medical education within each country. Unlike developing countries, European and developed countries enjoy considerable representation of their mother tongues in medical research production, reflecting the attention given to these languages. Disparities in native language usage in medicine between developed and developing countries highlight the significance of language in medicine and healthcare. It is critical to consider these language-related concerns and investigate methods for using native languages in medicine to close the gaps between language and medicine.

## Conflict of Interest

There are no conflicts of interest to declare.

## Authors Contribution Statement

AAH contributed to conceptualization, designing the manuscript, searching the literature, writing the original draft, and reviewing. JHJ contributed to searching the literature, analyzing and tabling PubMed database data, and writing the original draft. HKA contributed to searching the literature and writing the original draft. IMA contributed to the conceptualization and reviewing. All authors have critically reviewed and approved the final draft and are responsible for the content and similarity index of the manuscript.

## Figures and Tables

**Table 1.** Descriptive summary of native speakers worldwide and the number of publications on PubMed (From inception until December 2023).

**Language**	**Number of publications**	**Native speakers (in millions)**	**Language**	**Number of publications**	**Native speakers (in millions)**
English	31,699,206	380	Icelandic	1,915	0.3
German	905,322	76	(multiple Languages)	1,582	-
French	768,924	74	Lithuanian	1,233	3
Russian	703,527	148	Thai	648	21
Japanese	435,863	123	Slovenian	623	2
Spanish	384,183	486	Bosnian	611	3
Chinese	363,415	1,353	Arabic	322	383
Italian	308,390	64	Indonesian	225	44
(undetermined)	239,588	-	Persian	118	72
Polish	173,701	40	Catalan	89	4
Portuguese	117,173	236	Azerbaijani	83	22
Czech	89,341	10	Macedonian	59	2
Dutch	70,278	24	Latin	56	-
Danish	57,050	6	Albanian	51	6
Swedish	56,727	10	Armenian	39	3
Hungarian	44,842	12	Esperanto	36	0.001
Norwegian	41,119	5	Georgian	30	4
Romanian	28,351	25	Estonian	9	1
Bulgarian	22,519	7	Hindi	9	345
Croatian	19,814	5	Malay	9	82
Ukrainian	19,725	33	Latvian	7	2
Hebrew	18,183	6	Welsh	7	0.5
Finnish	17,882	5	Kinyarwanda	4	15
Serbian	17,238	10	Vietnamese	4	85
Slovak	15,962	5	Pushto	2	44
Turkish	11,363	84	Scottish gaelic	2	0.06
Korean	8,514	81	Maori	1	0.2
Greek, Modern	3,473	13	Sanskrit	1	-
Afrikaans	2,416	8			
